# Persistence of obligate intracellular pathogens: alternative strategies to overcome host-specific stresses

**DOI:** 10.3389/fcimb.2023.1185571

**Published:** 2023-05-22

**Authors:** Camille M. Riffaud, Elizabeth A. Rucks, Scot P. Ouellette

**Affiliations:** Department of Pathology and Microbiology, University of Nebraska Medical Center, Omaha, NE, United States

**Keywords:** persistence, obligate intracellular bacteria, *Chlamydia*, *Coxiella*, stringent response, tryptophan starvation, iron starvation, interferon-gamma

## Abstract

In adapting to the intracellular niche, obligate intracellular bacteria usually undergo a reduction of genome size by eliminating genes not needed for intracellular survival. These losses can include, for example, genes involved in nutrient anabolic pathways or in stress response. Living inside a host cell offers a stable environment where intracellular bacteria can limit their exposure to extracellular effectors of the immune system and modulate or outright inhibit intracellular defense mechanisms. However, highlighting an area of vulnerability, these pathogens are dependent on the host cell for nutrients and are very sensitive to conditions that limit nutrient availability. Persistence is a common response shared by evolutionarily divergent bacteria to survive adverse conditions like nutrient deprivation. Development of persistence usually compromises successful antibiotic therapy of bacterial infections and is associated with chronic infections and long-term sequelae for the patients. During persistence, obligate intracellular pathogens are viable but not growing inside their host cell. They can survive for a long period of time such that, when the inducing stress is removed, reactivation of their growth cycles resumes. Given their reduced coding capacity, intracellular bacteria have adapted different response mechanisms. This review gives an overview of the strategies used by the obligate intracellular bacteria, where known, which, unlike model organisms such as *E. coli*, often lack toxin-antitoxin systems and the stringent response that have been linked to a persister phenotype and amino acid starvation states, respectively.

## 1 Introduction

To survive adverse conditions encountered in their environment, including infection of a host, bacteria have adapted different survival strategies and stress responses. One of these strategies includes entry into an alternative growth mode or survival state called persistence. Persistence has been historically defined as the ability of a subset of the bacterial population to survive exposure to a bactericidal drug concentration. Characteristics of bacterial persistence include its transitory nature and its non-heritability by the progeny. Importantly, the surviving persister cells can return to normal growth when the stress is removed (Balaban et al., 2019). The term of ‘persisters’ was first used by Joseph Bigger in 1944 while describing a *Staphylococcus aureus* culture that could not be sterilized by high doses of penicillin. The phenotype was observed without the acquisition of mutations, suggesting a small minority of the bacterial population tolerated antibiotic exposure, possibly due to their reduced growth rate (Bigger, 1944). Antibiotic persistence is defined only for bactericidal antibiotics and the different types of antibiotic persistence, as well as guidelines for research on antibiotic persistence with bacteria cultivable in liquid medium, were recently defined in a consensus statement (Balaban et al., 2019).

The formation of persister cells is now appreciated as a common response by most bacterial species when encountering environmental stress including, but not limited to, antibiotic exposure. To survive inside their host cell, intracellular pathogens have evolved strategies to manage poor nutrient growth conditions, to escape from the immune system of the host, and to tolerate drug treatment. Intracellular persistence is observed when a pathogen is viable, but quiescent, within a host cell and can survive for long periods of time until the inducing stress is removed. For example, intracellular persistence of *Salmonella* is well-studied, and type II toxin-antitoxin (TA) systems are involved in *Salmonella* persistence after internalization by human macrophages (Helaine et al., 2014; Cheverton et al., 2016; Rycroft et al., 2018).

Obligate intracellular pathogens are also able to persist in their host cells with observations of ‘persisters’ having been made among members of the *Chlamydiaceae*, *Coxiellaceae*, *Anaplasmataceae*, and *Rickettsiaceae*. These pathogens are Gram-negative bacteria, responsible for a wide range of diseases in humans and animals worldwide. Among the *Chlamydiaceae*, three species are pathogenic to humans: *Chlamydia trachomatis*, *Chlamydia pneumoniae*, and the zoonotic pathogen *Chlamydia psittaci* (Elwell et al., 2016). *C. trachomatis* is the leading cause of bacterial sexually transmitted infections and infectious blindness, with an estimated one hundred million individuals infected worldwide each year (Rowley et al., 2019). Diseases resulting from *C. pneumoniae* and *C. psittaci* infections are community-acquired pneumonia or pneumonia transmitted by infected birds, respectively. *Coxiella burnetii* is the agent responsible for the zoonotic disease Q fever, first described in 1937 among abattoir workers (Derrick, 1983; Eldin et al., 2017). Q fever is difficult to diagnose because of the flu-like and non-specific symptoms of the acute form of the disease: high fever, severe headache, fatigue, and chills (Eldin et al., 2017). There are two primary species of the *Anaplasmataceae* family that cause significant disease in humans: *Anaplasma phagocytophilum* and *Ehrlichia chaffeensis*, responsible for the human granulocytic anaplasmosis and the human monocytotropic ehrlichiosis diseases, respectively (Salje, 2021). The *Rickettsiaceae* family comprises two genera including human pathogens: *Rickettsia* and *Orientia*. *Rickettsia* spp. are small coccobacilli and include the causative agents of Rocky Mountain Spotted Fever (*Rickettsia rickettsia*), Mediterranean spotted fever (*Rickettsia conorii*), and louse-borne typhus (*Rickettsia prowazekii* and *Rickettsia typhi*) (Salje, 2021). The genus *Orientia* contains a single species, the rod-shaped bacterium and causative agent of the severe human zoonotic disease scrub typhus, *Orientia tsutsugamushi* (Wongsantichon et al., 2020). The mechanisms underlying persistence of obligate intracellular bacteria are poorly understood. Here, we aim to present the current state of knowledge of persistence among the *Chlamydiaceae*, *Coxiellaceae*, *Anaplasmataceae*, and *Rickettsiaceae*.

## 2 General aspects of persistence in free-living bacteria

Chromosomal TA systems have been linked to bacterial persistence since the observation that mutations in the *hipA* gene of the *hipBA* TA system induce a high proportion of *E. coli* persistent bacteria and that the *dinJ-yafQ*, *mazEF*, *relBE*, and *yefM-yoeB* TA systems were highly expressed in the *E. coli* persistent population (Moyed and Bertrand, 1983; Keren et al., 2004; Shah et al., 2006). TA systems are widespread genetic loci that encode both a stable toxic protein, whose overexpression can lead to growth arrest or cell death, and a corresponding unstable antitoxin, which neutralizes the toxin’s activity during bacterial growth (Van Melderen and Saavedra De Bast, 2009). TA systems are classified into six types depending on the antitoxin’s nature and mode of action. While all the toxins are proteins, the antitoxins can be either noncoding RNAs (in type I and III systems) or low-molecular-weight proteins (in types II, IV, V, and VI) (Riffaud et al., 2020). In this section, we first discuss the type II TA system, for which the antitoxin neutralizes the toxin by protein-protein interaction. We then discuss the type I TA system, for which the antitoxin is a *cis* or *trans*-encoded antisense small RNA that interacts with the toxin-encoding mRNA by pairing, thereby inhibiting toxin mRNA translation and/or inducing its degradation (Riffaud et al., 2020).

**Figure 1:**
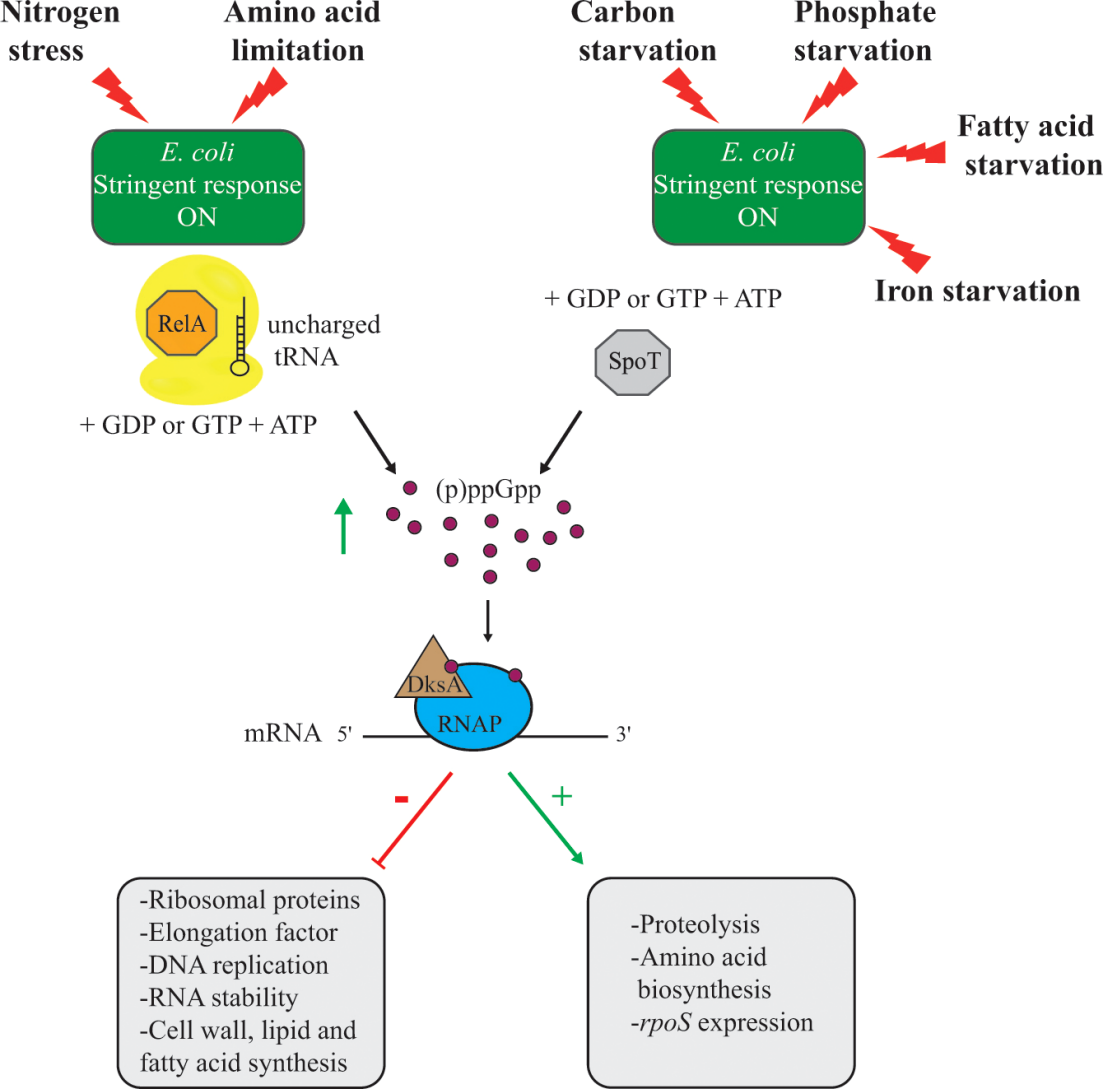
Illustration of the role of the stringent response in *E. coli* adaptation to various stressors. In *E. coli* the presence of an uncharged tRNA in the ribosome induces (p)ppGpp synthesis by RelA. In addition, carbon, phosphate, fatty acid, and iron starvation also lead to (p)ppGpp accumulation through SpoT. The accumulation of (p)ppGpp molecules in the *E. coli* cytoplasm induces the stringent response. The transcription factor DksA facilitates (p)ppGpp binding to the RNA polymerase (RNAP) to modulate gene expression.

Based on studies with *E. coli*, the proposed persister model included the stringent response and type II TA systems. The stringent response is a global regulatory program used by most bacteria in response to amino acid limitation. The ribosome-associated and monofunctional synthetase enzyme RelA is activated when an uncharged tRNA binds in the A site of the ribosome, leading to the synthesis of the guanosine pentaphosphate or guanosine tetraphosphate molecule (abbreviated (p)ppGpp; **Figure 1**) (Gourse et al., 2018). The signaling molecule ppGpp accumulates in the bacterial cytoplasm following exposure to amino acid limitation, or nitrogen stress (Brown et al., 2014; Fitzsimmons et al., 2018; Gourse et al., 2018). Furthermore, the activation of the bifunctional synthetase and hydrolase protein SpoT leads to ppGpp accumulation upon exposure of a wide range of stresses such as carbon starvation (Xiao et al., 1991; Meyer et al., 2021), phosphate starvation (Spira et al., 1995; Germain et al., 2019), fatty acid starvation (Battesti and Bouveret, 2006; Germain et al., 2019), or iron starvation (Vinella et al., 2005). The accumulation of ppGpp alters the expression of genes involved in amino acid biosynthesis and proteolysis, or global stress response (Hauryliuk et al., 2015) (**Figure 1**). Furthermore, the transcriptional factor DksA facilitates the binding of (p)ppGpp to the RNA polymerase, which further downregulates the transcription of rRNA operons and many genes encoding proteins involved in DNA replication, translation, cell wall, lipid, and fatty acid biosynthesis (Gourse et al., 2018) (**Figure 1**). Stochastic activation of the *hipBA* TA system was proposed to induce the *E. coli* stringent response followed by protease (specifically Lon) degradation of antitoxins, leading to activation of type II TA systems. Many of these toxins exhibit mRNase activity (e.g. RelE, YoeB, or MazF), shutting down global translation with subsequent persister cell formation (Fraikin et al., 2020). The work leading to this model has since been retracted and further questioned by recent work that challenged the findings (Shan et al., 2017; Goormaghtigh et al., 2018; Goormaghtigh and Van Melderen, 2019; Zalis et al., 2019). For example, in *S. aureus* and *E. coli*, deletion of type II TA systems did not impact the frequency of persister cells formed *in vitro*.

It has also been proposed that persister cells have low intracellular levels of ATP and that this may be a trigger for persister cell formation (Conlon et al., 2016; Shan et al., 2017). However, a recent study showed slow growth is sufficient to induce persistence of *Salmonella* without ppGpp, TA systems, or low ATP (Pontes and Groisman, 2019). In a recent study, Dewachter and colleagues proposed that, during the stationary phase of growth, protein aggregation and ATP depletion is enough to induce *E. coli* persistence. They also identified the GTPase ObgE as a persister protein promoting protein aggregation (Dewachter et al., 2021). Therefore, confusion remains in the persistence field of research and, even in model systems, efforts are still needed to characterize the molecular mechanisms governing persistence.

On the other hand, the type I TA systems (RNA antitoxin) likely function in some capacity in persister formation. In nutrient-starved *E. coli*, induction of the stringent response leads to an increase in translation of the Obg protein, which induces the expression of the type I toxin HokB (Verstraeten et al., 2015). The HokB toxin of the *hokB*/SokB system is then inserted in the *E. coli* membrane and induces pore formation, a decrease in membrane potential, and leakage of ATP (Wilmaerts et al., 2018). Altogether, this results in dormancy and tolerance to ofloxacin, an inhibitor of bacterial DNA replication that binds to DNA gyrase and topoisomerases II and IV, and tobramycin, an inhibitor of protein synthesis that prevents the formation of 70*S* ribosome complexes (antibiotics used for the persistence assays) (Verstraeten et al., 2015; Wilmaerts et al., 2018). However, HokB does not induce *E. coli* death. Resuscitation experiments of persister cells triggered by ofloxacin, with or without *hokB* induction, involved the removal of ofloxacin and measuring the single-cell lag time of persister cell outgrowth. These experiments showed that HokB-induced persister cells can resume growth, suggesting bacteria remain viable upon HokB expression (Wilmaerts et al., 2018).

Other studies showed that the TisB toxin of the *tisB*/IstR1 system in *E. coli* is induced during the SOS response, allowing bacterial persistence by decreasing the proton motive force, thus indirectly promoting tolerance to antibiotics such as β-lactams and fluoroquinolones by slowing growth (Dorr et al., 2009; Gurnev et al., 2012). The SOS response is a programmed DNA repair regulatory network, induced by stalled replication forks and DNA damage. The SOS response is controlled by the repressor LexA and the inducer RecA (Butala et al., 2009). Independently, using a *psulA::gfp* reporter, another study conducted by Goormaghtigh & Van Melderen characterized the entire persistence cycle of *E. coli* treated with ofloxacin and showed that sensitive and persister cells induce the SOS response similarly during the treatment, as SulA is found to be expressed in both (Goormaghtigh and Van Melderen, 2019). They also observed that persister cells present a maximum SOS induction at the time of recovery after ofloxacin treatment, with a peak of GFP fluorescence, and undergo massive cell filamentation (Goormaghtigh and Van Melderen, 2019). Therefore, SOS induction is not sufficient for persistence but may predispose bacteria to becoming persisters upon fluoroquinolone treatment.

In most publications, researchers have hypothesized that the persister phenotype results from toxin activation. However, a recent report in *S. aureus* demonstrated that the antitoxin SprF1 from the type I *sprG1*/SprF1 TA system can affect formation of persistent bacteria. SprF1 associates with translating ribosomes to reduce translation during growth, and this interaction is mediated through a purine-rich sequence of 6 nucleotides at the 5ʹ-end of SprF1 (Pinel-Marie et al., 2021). Furthermore, *sprG1*/SprF1 knock-out or deletion of the purine-rich sequence significantly decreased persister formation after ciprofloxacin or vancomycin treatment, demonstrating that SprF1 antitoxin function facilitates formation of *S. aureus* persisters (Pinel-Marie et al., 2021). Consequently, even antitoxins may have toxin-like effects under specific conditions.

Even in model bacteria like *E. coli* and *S. aureus*, the mechanisms leading to persistence appear to be challenging to identify and may imply different pathways depending on the persistence trigger. Many studies are conducted in test tubes and the reality of persistent infections may be more complex with other factors that may induce the persister state such as phagocytosis or an acidic environment (Helaine et al., 2014; Leimer et al., 2016). Nonetheless, growth arrest appears to be a key characteristic of persistence, and different stresses that impair essential functions necessary for bacterial growth increase antibiotic tolerance and persistence. This is also likely to be true for obligate intracellular bacteria where their growth is limited or stalled during stressful conditions.

## 3 Particularities of the intracellular lifestyle

To understand persistence in obligate intracellular bacteria, an appreciation for their unique biology must be gained. One consequence of the adaptation to the intracellular lifestyle is that obligate intracellular pathogens usually exhibit reduced genomes compared to free-living extracellular bacteria (pathogenic or otherwise). The intracellular lifestyle offers a stable environment in which to replicate and is, in many cases, more stable than extracellular environmental conditions where large shifts in temperature, pH, and nutrient availability can occur. Consequently, in evolving to obligate intracellular dependence, many pathogens have eliminated stress response elements, including those that were thought to play an important role in the formation of persister bacteria such as TA systems and the stringent response (see **Table 1**). Overall, it is likely that obligate intracellular bacteria have developed their own mechanisms to respond to stress and persist within their hosts.

**Table 1:** Presence or absence of stress response gene in human obligate intracellular pathogens compared to model free-living pathogens.

	TA system	General stress response			Amino acid starvation response				Iron starvation response	
		*obgE*	*rpoS/sigB*	*usp*	*relA*	*spoT*	*rsh*	*trpAB*	*ytgR*	*fur*
Obligate intracellular pathogens										
*Chlamydia* spp.	–	+	–	–	–	–	–	+^*^	+	–
*Coxiella burnetii*	–	+	+	+	+	+	–	+^**^	–	+
*Anaplasma* spp.	–	+	–	–	–	–	–	–	–	–
*Ehrlichia chaffeensis*	–	+	–	–	–	–	–	–	–	–
*Rickettsia* spp.	+	+	–	–	–	+	–	–	–	–
*Orientia tsutsugamushi*	–	+	–	–	–	+	–	–	–	–
Free living pathogen										
*Escherichia coli*	+	+	+	+	+	+	–	+	–	+
*Staphylococcus aureus*	+	+	+	–	–	–	+	+	–	+

(+) indicates the presence and (–) indicates the absence of specific genes.

*In human pathogenic species, the genes are present in *C. trachomatis* only.

**The genomic organization of the *trp* genes varies between *C. burnetii* isolates. The reference isolates NMRSA493 encodes an intact *trpA* but *trpB* and *trpF* are fused. In the case of the human chronic endocarditis isolates K and G, *C. burnetii* isolate K has the same organization of the *trp* genes than NMRSA493 whereas *C. burnetii* isolate K has fused *trpA* and *trpB* and an intact *trpF*.

The well-adapted human pathogen *C. trachomatis* has a genome of 1.04 Mbp with 895 open reading frames (ORFs) and few pseudogenes (Stephens et al., 1998). *C. trachomatis* has no annotated orthologs of *relA* or *spoT* and lacks identified TA systems. *C. burnetii*, meanwhile, has a genome of approximately 2 Mbp with 2,094 ORFs and 83 pseudogenes (Seshadri et al., 2003). 29 insertion sequence (IS) elements were also found in the genome of *C. burnetii*, suggesting high genomic plasticity and an ongoing evolutionary process to obligate intracellular dependence (Seshadri et al., 2003). Comparative genomic analysis revealed the presence of single elements of TA systems such as a HigA-like antitoxin or a RelE-like toxin among the pseudogenes within the *C. burnetii* genome, suggesting removal of TA systems during the reductive evolutionary process (Beare et al., 2009; Hemsley et al., 2019). However, *Coxiella* does possess *relA* and *spoT* orthologs (**Table 1**). Rickettsiales genomes differ in size and content. Their size ranges from 0.86 Mbp for *Neorickettsia sennetsuto* to 2.5 Mbp for *O. tsutsugamushi*. Interestingly, 49% of the *O. tsutsugamushi* genome is composed of repetitive elements (Salje, 2021). The presence of pseudogenes and repetitive elements indicates a genome reduction in progress likely linked to ongoing adaptation to obligate intracellular life. Among the repetitive elements, *Rickettsia* spp. encode several copies of *spoT* located on mobile genetic elements. Some copies are truncated and others encode a protein containing both the hydrolase and synthetase domains for (p)ppGpp catalysis and synthesis (Gillespie et al., 2012). The *O. tsutsugamushi* genome also possesses a complete *spoT* gene suggesting *O. tsutsugamushi* may produce (p)ppGpp (Nakayama et al., 2008; Gillespie et al., 2015). In the order Rickettsiales, TA systems have been identified in the genome of *Rickettsia* spp. only (Pandey and Gerdes, 2005; Gillespie et al., 2008; Audoly et al., 2011); (see **Table 1**).

A consequence of the reduction of genome sizes during evolution towards the intracellular lifestyle is the suppression of genes not needed for nutrient anabolic processes since these pathogens acquire essential nutrients from the host cell. This typically leads to multiple auxotrophies. For example, *Chlamydia* spp. are auxotrophs for tryptophan (Trp) and most other amino acids (Beatty et al., 1994), *C. burnetii* is auxotrophic for arginine, cysteine, histidine, leucine, lysine, phenylalanine, proline, tyrosine, threonine, tryptophan, and valine (Seshadri et al., 2003; Sandoz et al., 2016), and *O. tsutsugamushi* is auxotrophic for histidine and aromatic amino acids (Min et al., 2008). *R. prowazekii* and the *Anaplasmataceae* species can produce glycine, glutamine, and aspartate, but *E. chaffeensis* is only able to synthesize arginine and lysine (Dunning Hotopp et al., 2006). These amino acid auxotrophies highlight the dependence of obligate intracellular bacteria on the host cell to acquire nutrients, especially amino acids. This further suggests that limiting access to amino acids will have detrimental effects on these pathogens, which in most cases lack functional stringent responses. Therefore, how they respond to amino acid limitation is a question of fundamental interest.

The environment of intracellular bacteria differs if they reside within a pathogen containing vacuole or inside the cytoplasm of the host cell. Growth of *Chlamydia* spp., *C. burnetii*, and *Anaplasma* spp. occurs within pathogen containing vacuoles. This vacuole is called the inclusion for *Chlamydia*, the *Coxiella*-containing vacuole (CCV) for *C. burnetii*, and the morulae for *Anaplasma* spp (Moore and Ouellette, 2014; Eldin et al., 2017; Salje, 2021). These vacuoles confer protection against osmotic stress and the host immune system *via* the incorporation of host lipids and secreted bacterial effector proteins, which mimic host proteins in the membrane of the vacuole. Vacuole maturation is necessary to the success of vacuolar pathogens’ growth, and this comprises blocking fusion with lysosomes and redirecting host nutrients to the vacuole for most intracellular pathogens. An exception to this is *Coxiella*, which prefers the lower pH of the lysosomal compartment that is necessary for its growth. Regardless, these processes are directed by secreted bacterial effector proteins – typically mediated by type III (*e.g., C. trachomatis*) or IV secretion systems (*e.g., C. burnetii*, Rickettsiales) (Moore and Ouellette, 2014; Eldin et al., 2017; Salje, 2021). Living in a vacuole also implies more restricted accessibility of nutrients compared to organisms that grow within the cytosol, and these pathogen-containing vacuoles interact with various cellular compartments like the endoplasmic reticulum and Golgi apparatus to scavenge amino acids and lipids. Vacuolar pathogens need to import nutrients and molecules necessary to their growth across two membranes, suggesting their greater vulnerability to poor nutrient conditions that will trigger stress responses. The lifestyle of *Rickettsia* spp. and *O. tsutsugamushi* is different. These pathogens specifically lyse their phagosome to replicate within the cytoplasm of the host cell and usually have a higher replication rate than pathogens living in a vacuole due to the better supply of essential nutrients in the cytosol (Eisenreich et al., 2020).

Within their intracellular niche, intracellular bacteria hide from immune system effectors. Some have adapted to reduce or alter, or even delete, expression of bacterial markers recognized by the immune system such as the outer membrane lipopolysaccharide (LPS) or peptidoglycan. For example, no LPS was observed in *O. tsutsugamushi* or *Anaplasma* spp (Wongsantichon et al., 2020; Salje, 2021). Concerning peptidoglycan, only a transient peptidoglycan ring has been detected at the septum of dividing *C. trachomatis* (Liechti et al., 2014). Among the Rickettsiales order, *Rickettsia* spp. produce a complete peptidoglycan sacculus, and a minimal peptidoglycan-like structure was recently identified in *O. tsutsugamushi* (Pang and Winkler, 1994; Atwal et al., 2017). *A. phagocytophilum* is unable to produce peptidoglycan (Salje, 2021). Among the vacuolar pathogens, *C. burnetii* faces very adverse growth conditions because its target host cell in mammals is a macrophage. *C. burnetii* replicates in an exceptionally stressful environment and encodes a significant number of basic proteins suggesting a function in buffering the acidic environment of the CCV (Eldin et al., 2017). Interestingly, the infectious but non-replicating form of *C. burnetii*, the spore-like small cell variant (SCV), synthesizes a very thick peptidoglycan layer conferring resistance to osmotic and mechanical insults to the SCV and enhancing its environmental stability (Eldin et al., 2017).

Independently of their lifestyle, persistence of bacterial pathogens is usually associated with chronic infections, symptomatic or not, therapeutic failure, and long-term sequelae for the patients. Asymptomatic persistent infections are typically undiagnosed and represent a burden for the host that can also serve as a reservoir of infection to spread the disease, highlighting the clinical relevance of persistent infections (Fisher et al., 2017). *In vitro* persistence is characterized for only a few obligate intracellular species, however chronic or relapsing infections have been described for all of them. The ability of these pathogens to persist *in vivo* may represent both an important survival strategy and a critical reservoir for spread to other individuals. In this work, we describe the most recent advances in persistence of obligate intracellular bacteria such as the *Chlamydiaceae*, *Coxiellaceae*, *Anaplasmataceae*, and *Rickettsiaceae* and how their intracellular lifestyle may influence their unusual mechanisms to induce persistence. At a molecular level, persistence is best characterized in *Chlamydia*, highlighting potential avenues of investigation in other obligate intracellular bacteria.

## 4 The *Chlamydiaceae* and the persistence phenotype

### 4.1 Clinical relevance

The two main pathogenic species to humans, *C. trachomatis* and *C. pneumoniae*, produce persistent infections. It is estimated that >70% of *C. trachomatis*-induced endocervical infections are asymptomatic and can persist for prolonged periods of time in the absence of treatment (Stamm, 1999; Satterwhite et al., 2013). This suggests that, each year, infections are significantly underdiagnosed. Persistent *C. trachomatis* infections can result in the activation of the host immune response and chronic inflammation. This chronic inflammation is likely responsible for tissue damage and scarring, the long-term sequelae of which include pelvic inflammatory disease, ectopic pregnancy, and infertility in women, and epididymitis or urethritis in men (Brunham and Rey-Ladino, 2005; Darville and Hiltke, 2010). Chlamydial persistence is associated with aberrant organisms (aRBs – see more below), and *C. trachomatis* aRBs have been observed in human endocervix samples, suggesting the presence of persistent *Chlamydia* in patients (Lewis et al., 2014). In addition, chlamydial antigens, for example DNA or RNA, can be detected in clinical samples, even after antibiotic treatment and culture negativity. Indeed, *C. trachomatis* DNA was found in fallopian tube tissue biopsies from a woman culture-negative for *Chlamydia* but suffering from post-infectious infertility, a sequelae associated with persistent infection (Patton et al., 1994). In another study, Suchland et al. (2017) analyzed whole-genome sequences of clinical samples collected from women over several years. Remarkably, their results indicated that the same *C. trachomatis* strain persisted for 3-5 years in the female genital tract. Furthermore, the genome of that strain was remarkably stable, without the accumulation of major mutations linked with antibiotic therapies, suggesting a persister state (Suchland et al., 2017). *C. pneumoniae* is a respiratory pathogen responsible for approximately 10% of community-acquired pneumonia. Persistent *C. pneumoniae* infections are associated with airway inflammation and chronic lung diseases such as chronic obstructive pulmonary disease and asthma (Kuo et al., 1995; He et al., 2010). In an *in vivo* model of *C. pneumoniae* infections, it has been observed that cortisone can induce *C. pneumoniae* reactivation in the lungs of the animals. This indicates that the immune system does not completely eradicate the bacteria that persist in the infected tissues (Malinverni et al., 1995; Laitinen et al., 1996). Multiple studies have associated *C. pneumoniae* infection of macrophages with foam cell formation and atherosclerosis, and the organism has been isolated from coronary samples (Gieffers et al., 1998; Byrne and Kalayoglu, 1999; Maass et al., 2000; Gieffers et al., 2001). As atherosclerotic plaques develop over many years and are slow growing, persistent *C. pneumoniae* infection may contribute to coronary artery disease.

Most *Chlamydia* strains are susceptible to azithromycin and doxycycline, two antibiotics used to treat infections. However, very low doses of doxycycline, 0.03–0.015 mg/L for 72 hours, can be a trigger for a cell culture model of *C. trachomatis* persistence in HeLa cells, where organisms could continue growth after removal of these low doses of doxycycline (Marangoni et al., 2020). However, further work is needed to determine whether doxycycline can induce persistence *in vivo*. Recently, it was observed that among a group of 840 pregnant women tested positive for *C. trachomatis* and treated with azithromycin, 13.6% were tested positive on all 3 tests performed during their pregnancy suggesting treatment failure and recurrent or persistent infections during pregnancy (Dionne-Odom et al., 2020).

### 4.2 Biology

All members of the *Chlamydiaceae* family undergo a biphasic developmental cycle that alternates between two distinct morphological forms: a small extracellular and infectious form called the elementary body (EB) and a larger intracellular replicative form called the reticulate body (RB) (Elwell et al., 2016). This developmental cycle varies in timing depending on the *Chlamydia* species (Wyrick, 2010). Briefly, following entry into a host cell through endocytosis or phagocytosis, EBs reside in the inclusion where they differentiate into replicative RBs. After several cycles of division by a polarized budding mechanism, RBs undergo secondary differentiation into EBs, which are released after lysis of the host cell or by an extrusion mechanism (Abdelrahman and Belland, 2005; Abdelrahman et al., 2016).

### 4.3 Persistence

In *Chlamydia*, persistence is defined as a viable but non-infectious reversible state of the chlamydial developmental cycle characterized by a block in cell division, by morphologically aberrant enlarged RB size of ≈2 to 10 μm, and by a reduction/elimination in progeny production (Wyrick, 2010). Like persister cells in model organisms, chlamydial persistence reflects stalled or limited growth. Unlike persisters in model bacteria, where only a subset of organisms undergoes this transition, chlamydial persistence is typically uniform across the culture based on consistent aberrant morphology. This again highlights the unique mechanism(s) this pathogen uses to persist. Chlamydial persistence can be triggered in cell culture models by a large variety of stimuli including the host cytokine interferon gamma (IFN-γ) (Beatty et al., 1993), starvation of tryptophan (Beatty et al., 1994) or specific amino acids (Coles et al., 1993), chelation of iron (Raulston, 1997), heat shock (Kahane and Friedman, 1992; Huston et al., 2008), certain antibiotics (Matsumoto and Manire, 1970; Phillips Campbell et al., 2012; Kintner et al., 2014; Hatch and Ouellette, 2020; Marangoni et al., 2020; Chen et al., 2021), infection of monocytes or macrophages (Koehler et al., 1997; Nettelnbreker et al., 1998; Gracey et al., 2013), and coinfection with viruses (Deka et al., 2006; Borel et al., 2010; Koster et al., 2022). Small inclusions containing enlarged *Chlamydia* with aRBs were observed after penicillin exposure, first in *Chlamydia muridarum* and *Chlamydia felis* in 1950, and later for *C. trachomatis* and *C. psittaci* (Weiss, 1950; Hurst et al., 1953; Tamura and Manire, 1968). Normal morphology was rescued after penicillin removal suggesting *Chlamydia* had entered a persistent growth state similar to other bacterial persisters (Matsumoto and Manire, 1970). During a natural infection, *Chlamydia* is likely exposed to two main persistence triggers: the inflammatory cytokine IFN-γ and iron starvation. We will focus on persistence triggered by these stresses in the subsequent sections, as these are the most biologically relevant stresses for *C. trachomatis*, especially in the case of asymptomatic infections. Importantly, both are linked to nutritional stress that stalls growth of the pathogen.

#### 4.3.1 IFN-γ-mediated persistence

**Figure 2:**
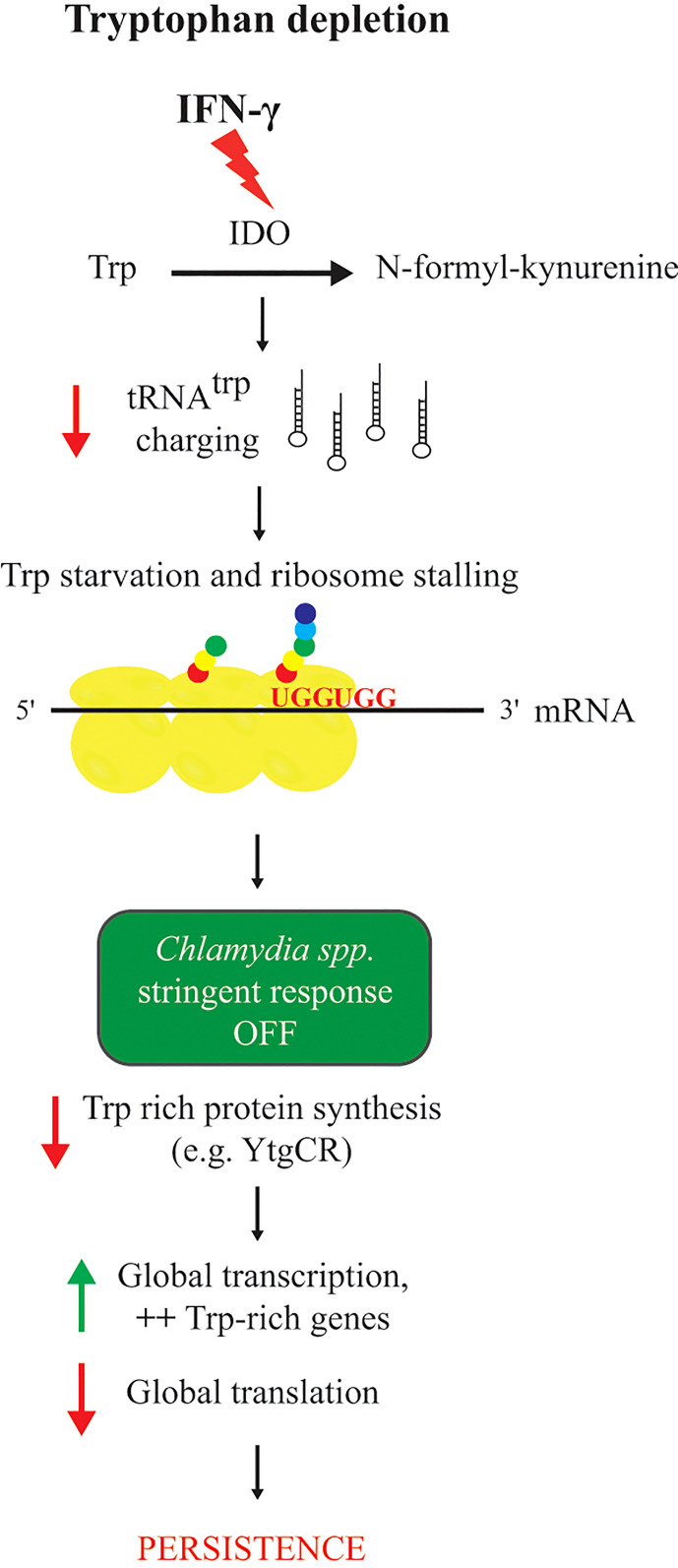
Response of chlamydial species to tryptophan starvation mediated by IFN-γ. Red arrows indicate a decrease and green arrows indicate an increase of a cellular function. IFN-γ is the trigger for Trp catabolization to N-formyl-kynurenine by the IDO enzyme, resulting in a Trp depleted environment, a decrease of tRNA^trp^ charging, and stalling at Trp codons (UGG). Unable to produce (p)ppGpp because *relA* or *spoT* are absent from its genome, *Chlamydia* spp. respond to Trp starvation mediated by IFN-γ by decreasing the production of Trp containing proteins (e.g. YtgCR), then by increasing transcription, more specifically for Trp-rich genes, and finally by a decrease of global translation leading to persistence.

How *Chlamydia* responds to IFN-γ exposure is one of the most studied persistence models among all obligate intracellular bacteria. IFN-γ is a pro-inflammatory cytokine and involved in the primary host response against endocervical *C. trachomatis* infections (Aiyar et al., 2014). IFN-γ-mediated persistence is particularly relevant in the context of chlamydial persistent infection in which the pro-inflammatory environment generated by asymptomatic infections is responsible for the long-term sequelae of chronic infections (Darville and Hiltke, 2010). IFN-γ induces Trp deprivation in human cells through activation of the expression of the enzyme indoleamine-2,3-dioxygenase (IDO; **Figure 2**). Because pathogenic *Chlamydia* species are auxotrophic for Trp, this low Trp environment induces the development of noninfectious atypical chlamydial forms, aRBs, within smaller inclusions (Beatty et al., 1994). Sensitivity of the chlamydial species infecting humans to IFN-γ mediated Trp depletion depends in large part on their growth rate. Faster growing species and serovars, like *C. trachomatis* serovar L2, typically require pretreatment of the host cell with IFN-γ (Morrison, 2000). This ensures an adequate Trp limiting environment when the pathogen is entering its logarithmic growth phase. *In vivo*, surrounding uninfected cells may become activated by IFN-γ produced from responding immune cells such that, once an infected cell lyses, the next cycle of infection is in the presence of “pre-treated” cells. In accordance with persistence reversibility, infectious progeny (meaning generation of new EBs from secondary differentiation) are produced when IFN-γ is removed and the Trp pool of the host cell is regenerated (Beatty et al., 1995). However, severe Trp limitation, where free Trp concentrations are effectively 0, can prevent induction of persistence as well as reactivation (Leonhardt et al., 2007), and this is likely tied to the timing of Trp starvation in relation to the developmental cycle. Only the RB can become an aRB, and if the EB cannot differentiate to the RB before Trp starvation, then it may not be able to persist for extended times.

Subsequent work has characterized IFN-γ-mediated persistence at the transcriptomic and proteomic levels. Intuitively, the hypothesis driving studies into chlamydial transcriptional responses during persistence posited that *Chlamydia* enacted a global “regulon” to respond to this stress state, similar to model bacteria using, for example, the alternative sigma factor RpoS (also called σ^S^ or σ^38^; **Table 1**) (Gottesman, 2019). Several targeted transcriptional studies measuring transcriptional changes in *Chlamydia* during IFN-γ-mediated persistence appeared to support this (Byrne et al., 2001; Slepenkin et al., 2003; Polkinghorne et al., 2006). Additionally, work from the McClarty lab also demonstrated the specific induction of the *C. trachomatis* TrpBA synthase in response to low Trp conditions after derepression of the TrpR regulator (Wood et al., 2003; Carlson et al., 2006). Further support for a “persistence regulon” came from work conducted by Belland and colleagues using microarray analyses. The microarray technique revealed global changes in the *C. trachomatis* serovar D transcriptome in an *in vitro* model of IFN-γ-mediated persistence with infected HeLa cells. By comparing *C. trachomatis* transcript profiles between cultures treated or not with IFN-γ, and at different times of exposure representative of different stages of the developmental cycle, Belland et al. suggested the induction of a “persistence stimulon” allowing *C. trachomatis* to survive (Belland et al., 2003a).

However, all of these studies generally took two approaches to measure transcriptional changes. In the first instance, transcripts of 16*S* rRNA or another “housekeeping” gene were used to normalize transcript data as is typically performed in other bacterial systems. This normalization strategy assumed that these genes remained constant during persistence and were proportional to the bacteria. Because of its unique developmental cycle including two morphological forms, gene expression in *Chlamydia* is temporal, allowing each gene to be expressed when its function is needed (Belland et al., 2003b). As a consequence, a housekeeping gene whose transcript level is the same throughout the developmental cycle of *Chlamydia* does not exist (Ouellette et al., 2005). In the second instance, these early studies typically compared untreated culture conditions to the IFN-γ treated cultures at 24- or 48- hours post-infection for *C. trachomatis* or *C. pneumoniae*, respectively, a time in which RBs are differentiating to EBs in untreated conditions. In 2006, work from the Byrne lab demonstrated that both of these strategies were flawed. For example, 16*S* rRNA transcripts were measured during IFN-γ mediated persistence in *C. pneumoniae* and normalized to genomic DNA levels, the template of transcription. 16*S* rRNA transcripts should remain proportional to genome copies over time and during persistence if 16*S* rRNA is an appropriate normalizer for transcript levels. However, 16*S* rRNA transcripts increased in proportion to the genome copy during IFN-γ treatment, consistent with their lack of a stringent response (i.e. a relaxed phenotype). These data indicated that the template of transcription is more reliable and appropriate for normalizing transcript data, particularly during persistence (Ouellette et al., 2006). Transcriptional changes of the nascent RB as it becomes persistent were measured longitudinally during those experiments rather than comparing a population of RBs differentiating to EBs (as occurs at later time points during the developmental cycle) to a persistent culture.

Combining these two modifications to normalize and assess transcript data during persistence revealed that depriving *C. pneumoniae* of Trp by IFN-γ treatment does not induce the expression of a specific regulon. Using microarray analyses during IFN-γ-mediated persistence and normalization of the data to genomic DNA demonstrated that *C. pneumoniae* transcription was globally increased. However, chlamydial translation was globally decreased, as assessed by metabolic labeling of the chlamydial protein pool (Ouellette et al., 2006) (**Figure 2**). The fact that Trp is incorporated in approximately 80% of chlamydial proteins with an average Trp content per protein of 0.96% for *C. pneumoniae* may explain the global decrease in translation (Ouellette et al., 2016). It was hypothesized that, given the lack of a stringent response to trigger persistence in these organisms, *Chlamydia* might respond transcriptionally by increasing the transcription of genes rich in Trp codons. To investigate this, RT-qPCR analysis was used to measure the transcript levels of targeted genes selected for their Trp codon-content (free (0%), poor (between 0 and 0.96%), or rich (above 1.2%)). With this approach, transcripts of Trp codon-rich genes were observed to increase whereas transcripts of Trp codon-free or Trp codon-poor genes were decreased or unchanged during IFN-γ-mediated Trp starvation (Ouellette et al., 2016) (**Figure 2**). This trend was also observed when analyzing the microarray data based on amino acid codon content of the specific transcripts such that Trp-codon rich transcripts, as a group, were disproportionately increased. Interestingly, transcripts of operons and large genes were disproportionately decreased, suggesting that ribosome stalling during Trp starvation leads to premature transcript termination. A function for the transcriptional terminator Rho in this process was demonstrated to cause this effect (Ouellette et al., 2018).

Recently, RNAseq during IFN-γ-mediated persistence of *C. trachomatis* serovar L2 was used to compare its responses to another Trp auxotrophic pathogen, *Streptococcus pyogenes* (Ouellette et al., 2021b). It was hypothesized that the chlamydial response to Trp starvation was due to its lack of a stringent response, thus chlamydial transcriptional changes were compared to those of wild-type and stringent response mutant (*relA*-) strains of *S. pyogenes*, assessed as a function of their respective amino acid content. Surprisingly, *S. pyogenes* strains responded in a similar manner to Trp starvation as *Chlamydia*, irrespective of the stringent response. Importantly, these effects were not due to differential stabilization or protection of Trp codon-rich transcripts from ribosome stalling, as previously noted (Ouellette et al., 2018). These data suggest that increasing transcription of Trp codon-rich genes in comparison to Trp codon-poor genes may be a conserved strategy for Trp auxotrophs to respond to Trp starvation. Further work is necessary to understand the mechanistic underpinnings of this response to Trp starvation. Overall, these data indicate that transcriptional studies of *Chlamydia* during IFN-γ-mediated persistence cannot be extrapolated to infer the physiological state of persistently growing organisms.

**Figure 3:**
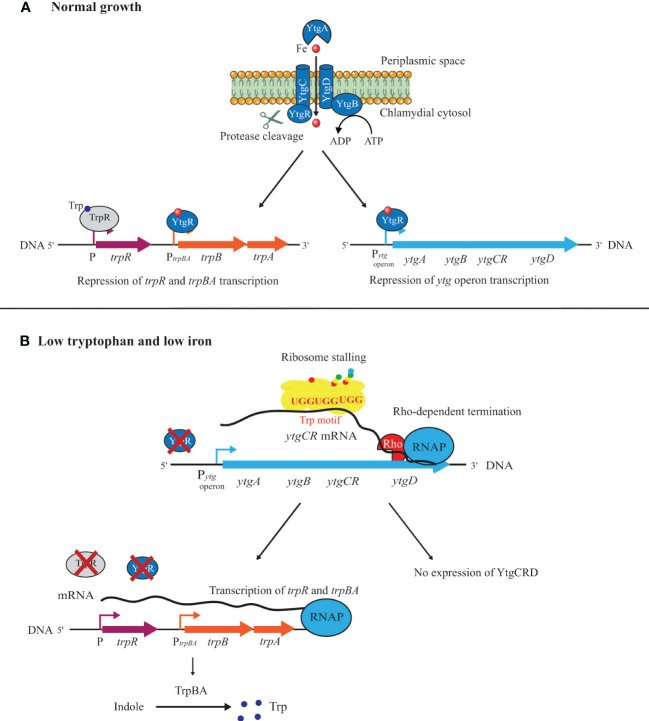
Model of tryptophan and iron-dependent expression of the operons *ytgABCRD* and *trpBA*. **(A)** Under normal growth, the presence of Trp in the environment allows ribosomes to read through the Trp motif of the *ytgCR* mRNA to complete its translation. After cleavage from YtgC and the binding of an iron molecule, YtgR functions as a transcriptional repressor for the *ytg* operon and the *trpBA* operon. In these conditions, the expression of TrpBA is downregulated by the TrpR and YtgR repressors. **(B)** Under Trp starvation, the ribosome stalling at the WWW motif of *ytgCR* and the Rho-dependent termination of transcription affect the translation of YtgCR. Under low iron conditions, YtgR does not bind its repressor sites in promoter regions. These low Trp and low iron conditions of growth allow the removal of the TrpR and YtgR repressors from the *trpR* and *trpBA* promoters allowing their transcription. In the presence of indole, Trp can be produced *via* the activity of the TrpBA synthase.

How then can we assess the physiological state of *Chlamydia* given the disconnect between transcription and translation during IFN-γ-mediated persistence? Effectively, the phenotype is driven primarily from the protein content of the organism, therefore, proteomic assessments should be more informative to our understanding of persistence. Bonner et al. (2014) proposed that, during their evolution, a decrease in Trp content in specific proteins within specific pathways (e.g. menaquinone synthesis) renders these pathways more resistant to Trp starvation mediated by IFN-γ to allow *Chlamydia* to survive during persistence (Bonner et al., 2014). A corollary of this hypothesis is Trp-codon richness in specific transcripts may render certain genes more susceptible to Trp starvation, and this could help trigger persistence. For example, expression of the YtgCR protein, which contains a triple Trp motif (i.e. WWW), is negatively impacted during Trp starvation (**Figure 3**). Pokorzynski et al. (2020) mutated this motif to YFF and showed that expression of this mutant isoform was recovered during Trp starvation in comparison to the wild-type protein (Pokorzynski et al., 2020). Interestingly, the YtgCR protein is involved in the response to iron limitation, thus potentially linking Trp starvation and iron limitation (**Figure 3**). In this scenario, the inability to translate the YtgR repressor protein activates the *ytg* regulon (see more below for persistence induced by iron limitation).

In a recent study, Riffaud et al. hypothesized that chlamydial cell division is blocked during Trp starvation-mediated persistence by the inability to translate Trp-rich cell division proteins. Compared to *E. coli* and other obligate intracellular pathogens, some *C. trachomatis* L2 cell division genes encode proteins enriched in Trp residues (e.g. FtsI/Pbp3, MraY, RodA; see **Table 2**). The Trp content significantly decreased the expression of two wild-type division-related proteins containing a WW motif (RodZ and CTL0293) during persistence induced by Trp starvation. On the contrary, isoforms lacking Trp codons were still expressed in the same conditions (Riffaud et al., 2023). By looking more broadly at cell division and PG-synthesis proteins in *Chlamydia*, immunofluorescence data demonstrated that the Trp-poor protein MurG and the Trp-neutral protein FtsL were still expressed during persistence, while the expression of the Trp-enriched proteins Pbp2, RodA, FtsI/Pbp3, and MraY was significantly reduced (Riffaud et al., 2023). Overall, this study provides a mechanistic explanation for how cell division is inhibited in *Chlamydia* during Trp starvation conditions and, together with the Pokorzynski et al. study, underscores the link between the Trp content of a protein and its likelihood to be translated.

**Table 2:** Tryptophan content in division and peptidoglycan synthesis proteins in human obligate intracellular pathogens compared to *E. coli*.

SpeciesProtein	*Escherichia coli*	*Chlamydia trachomatis*	*Coxiella burnetii*	*Anaplasma phagocytophilum*	*Ehrlichia chaffeensis*	*Rickettsia* spp.	*Orientia tsutsugamushi*
**AmiA**	1	1	5	–	–	–	–
**FtsA**	2	–	2	2	0	1	3
**FtsB**	2	4, **WW** (HP)	1	–	–	0	0
**FtsE**	1	(HP)	0	0	0	0	0
**FtsI/Pbp3**	6	6, **WW**	7	–	–	7	1
**FtsK**	19	7	7	4	2 (HP)	5	2
**FtsL**	1	1	2	–	–	1	0
**FtsN**	5	–	–	–	–	–	–
**FtsQ**	7	2	5	7	5 (HP)	3	4
**FtsW**	10	4	8	-*	–	7	6, **WW**
**FtsX**	4	0	5	2	1	1	–
**FtsZ**	0	–	0	0	1	1	1
**MraY**	10	8, **WW**	7	–	–	4	4
**MraW**	3	3	3	1	1	1	0
**MreB**	0	0	0	2, **WW**	–	0	0
**MreC**	1	6	2	2	–	0	0
**MurA**	2	5	2	-*	3	1	1
**MurB**	7	0	3	3	–	3	3
**MurC/DdlA**	1	7	2	-*	–	0	0
**MurD**	3	2	3	-*	–	2	2
**MurE**	7	1	1	-*	–	0	0
**MurF**	5	4	1	-*	–	2	2
**MurG**	9	1	5	-*	–	1	1
**MurJ**	9	9	12	5	–	1	0
**Pbp2**	10, **WW**	13	7	-*	–	6	7
**RodA**	8	8, **WAW**	9	-*	–	3	4
**RodZ**	6, **WWW**	4, **WW**	4, **WW**	–	–	0	–
**ZipA**	1	–	1	–	–	–	–

(–) indicates the absence of specific genes based on genomic context.

(HP) indicates the presence of a putative protein.

Tryptophan motifs are indicated in bold.

*The sequence of these proteins is not available for *A. phagocytophilum*. In *A. marginale*, the tryptophan content is 6 for FtsW, 2 for MurA, 3 for MurC/DdlA, 6 for MurD, 3 for MurE, 3 for MurF, 1 for MurG, 4 for RodA, and 7 for Pbp2, without any Trp codon.

Various studies have assessed protein expression during IFN-γ-mediated persistence. However, given the limited biomass of persistent chlamydial cultures as compared to the host cell biomass, such studies are challenging when implemented at the proteomic level. Nevertheless, in Beatty et al.’s original characterization of persistence, western blots were used to demonstrate that *C. trachomatis* serovar A Hsp60 levels remained constant whereas major outer membrane protein (MOMP) and OmcB, an EB-specific protein, levels were reduced (Beatty et al., 1993). Of note, Hsp60 contains zero Trp residues. These initial observations were confirmed and extended by Shaw et al. (1999), who observed a correlation between IFN-γ treatment and decreased levels of MOMP expression in *C. trachomatis* serovar A, but not *C. trachomatis* serovar L2 (Shaw et al., 1999). Using high-resolution two-dimensional immobilized pH gradient polyacrylamide gel electrophoresis (2D-PAGE) in combination with pulse labeling with [^35^S]methionine, they also observed up-regulation of proteins in *C. trachomatis* serovars A and L2. These included the two putative subunits of the Trp synthase, presumably induced to adapt to the Trp-depleted environment of the host cell (Shaw et al., 1999). In *C. pneumoniae*, increased levels of proteins involved in stress response, nucleotide and amino acid biosynthesis, DNA replication, transcription, translation, glycolysis, type III secretion, and cell envelope were observed during IFN-γ-mediated persistence (Molestina et al., 2002; Mukhopadhyay et al., 2006). However, these results are not consistent with recent comparative proteomic investigations conducted in *C. trachomatis* serovar D, possibly indicating species-specific differences. During IFN-γ-mediated persistence of *C. trachomatis* serovar D, Ostergaard and colleagues demonstrated that the proteomes of RBs and aRBs were broadly similar, with the exception of the two subunits of the Trp synthase TrpA and TrpB that were present at very high levels in aRBs (Ostergaard et al., 2016). This suggests an attempt by *C. trachomatis* to synthesize Trp from indole, the substrate of the Trp synthase (Shaw et al., 1999; Wood et al., 2003). Interestingly, it has been proposed that *C. trachomatis* genital serovars can scavenge indole from the normal flora to resist IFN-γ-mediated Trp limitation (discussed further below). Furthermore, aRBs were found to express lower levels of proteins with high Trp content (Ostergaard et al., 2016), which is consistent with the hypothesis proposed by Ouellette et al. where ribosomes stalling at Trp codons decrease global translation to trigger persistence (Ouellette et al., 2016).

From a clinical point of view, the burden of persistent chlamydial infections derives from the development of long-term sequelae by the patients, especially women, and on the relapse of infections. Infection relapse implies the reactivation of persistent bacteria to active growth. Indeed, the restoration of the Trp pool in the host cell is necessary to restore persistent *Chlamydia* to a normal developmental cycle (Beatty et al., 1995). In this pioneering work on chlamydial reactivation, Beatty et al. provided evidence that replacement of IFN-γ-treated medium by fresh IFN-γ-free medium is necessary for recovery of infectivity (EBs production) from aberrant chlamydial forms. Furthermore, removal of IFN-γ, and recovery of infectious *Chlamydia*, was accompanied by an increase of MOMP and the 60-kDa outer envelope OmcB protein synthesis and a reorganization of the aRBs to morphologically typical developmental forms (Beatty et al., 1995). An enzyme converting indole or serine into Trp, called Trp synthase, is expressed by *C. trachomatis* but not by *C. pneumoniae*. Remarkably, only the genital serovars of *C. trachomatis* encode functional *trpA* and *trpB* genes coding for the Trp synthase, in which the enzymatically dead TrpA functions as a scaffold for TrpB to form the functional Trp synthase complex (Fehlner-Gardiner et al., 2002; Caldwell et al., 2003; Muramatsu et al., 2016). *C. trachomatis* is unable to produce indole and scavenges this substrate from its environment. Interestingly, women with bacterial vaginosis, for whom the incidence of *C. trachomatis* infection is high, frequently possess a microbiome enriched with bacterial species containing indole producers like *Prevotella* spp (Brotman et al., 2010; Muramatsu et al., 2016).. This suggests that *C. trachomatis* genital serovars are dependent on the presence of indole-producer bacteria in the lower genital tract to circumvent Trp depletion mediated by IFN-γ (Aiyar et al., 2014). However, this implies that, as *Chlamydia* ascends the female genital tract, it will eventually enter a “sterile” area devoid of indole-producers. Consequently, *Chlamydia* in these areas will be susceptible to IFN-γ-mediated Trp limitation. Persistent chlamydiae may thus lead to chronic inflammation in these sites and subsequent sequelae like pelvic inflammatory disease.

As one strategy to identify factors important for chlamydial survival during IFN-γ-mediated Trp starvation, chemically mutagenized chlamydial strains have been used. For example, *C. trachomatis* L2 mutants unable to resume growth from IFN-γ-mediated persistence were identified. Despite the addition of indole in the medium, the mutant strains failed to produce Trp. Using a GFP-expressing library of chemically mutagenized strains of *C. trachomatis*, non-synonymous mutations in *trpB*, CTL0225 (encoding a hypothetical membrane protein), and the chlamydial homolog of the *E. coli cysJ*, CTL0694 (encoding a hypothetical oxidoreductase with orthologs present in all sequenced *Chlamydia* spp.), were identified in strains defective for recovery from IFN-γ-mediated persistence (Muramatsu et al., 2016). Another group identified the gene *ptr* encoding a putative protease required for recovery from IFN-γ-mediated persistence. Reduced infectious progeny production and impaired genome replication upon removal of IFN-γ were observed in a *ptr*-null strain, suggesting that strain is unable to efficiently return to a normal developmental cycle (Panzetta et al., 2019). Further mechanistic studies are needed to investigate the involvement of these proteins in reactivation.

Future studies investigating IFN-γ-mediated persistence will ideally leverage newly developed genetic tools to inducibly overexpress or knockdown specific targets during amino acid starvation (Bauler and Hackstadt, 2014; Ouellette et al., 2021a). To this end, we leveraged characterized bacterial tRNA synthetase inhibitors as a means to circumvent the need for treating host cells with IFN-γ, which often requires pre-treating the host cells to effectively induce persistence in *Chlamydia* – particularly faster growing serovars and species. This can lead to heterogeneity between labs that can make comparisons between studies challenging. We demonstrated that blocking tryptophanyl-tRNA synthetase using indolmycin effectively mimicked IFN-γ-mediated persistence (Hatch and Ouellette, 2020), to reflect the translation-associated effects of Trp starvation. Therefore, future studies should be able to combine genetic tools with these inhibitors to explore the function of specific proteins in the development of or reactivation from IFN-γ-mediated persistence.

#### 4.3.2 Iron deprivation-mediated persistence

Persistence mediated by iron-deprivation of *Chlamydia* is clinically relevant considering the physiology of the female genital tract. The lactoferrin protein is expressed at mucosal sites as part of the innate immune response to prevent accumulation of free iron. Indeed, lactoferrin produced by epithelial cells and neutrophils was demonstrated to have a bacteriostatic effect linked to its iron-binding activity, its inhibitory effect on bacterial adhesion and cell invasion, and its ability to induce bacterial lysis (Jenssen and Hancock, 2009; Valenti et al., 2018). An increase of interleukin-1β, interleukin-6, and interleukin-8 cytokine levels was observed in women with vaginosis (abnormal vaginal microbiome). This leads to an increase of lactoferrin concentration released by neutrophils recruited by these pro-inflammatory cytokines and presumably a decrease in iron availability owing to lactoferrin’s iron-binding activity. Furthermore, lactoferrin is strongly increased in lower genital tract mucosal fluid from women infected by *Neisseria gonorrheae*, *C. trachomatis*, and *Trichomonas vaginalis* infections, suggesting that these pathogens may be exposed to iron-limiting conditions (Valenti et al., 2018). *Chlamydia* acquires iron from the host, likely through an interaction with the slow transferrin recycling pathway (Ouellette and Carabeo, 2010), since they lack any identified siderophores, thus limiting the availability of this essential nutrient is detrimental to the bacterium.

In cell culture models, iron chelators can be used to sequester free iron. In 1997, the first study demonstrated that treatment of *C. trachomatis* serovar E-infected cells with desferrioxamine mesylate (DFO) induces aberrant and morphologically enlarged RB formation, with a delayed maturation and a significant reduction of EB production (Raulston, 1997). Moreover, the addition of iron-saturated holotransferrin (hTf) to the cell culture medium rescues EB production. hTf is an iron transport protein with two binding sites for Fe^3+^. The binding of iron is reversible and addition of hTf to the cell culture medium will increase iron availability. Thus, the phenotype associated with iron-deprivation is reversible, which is a hallmark of persistence (Raulston, 1997). Similarly, in *C. pneumoniae* and *C. psittaci*, iron deprivation also results in smaller inclusions with abnormal chlamydial morphology and a decrease in infectious progeny (Al-Younes et al., 2001; Freidank et al., 2001; Goellner et al., 2006). Interestingly, *Chlamydia* species did not respond equivalently to the treatment of HEp-2 infected cells by DFO, which had more potent effects on *C. pneumoniae* than *C. trachomatis* as characterized by smaller inclusions and reduced infectious EB production (Pokorzynski et al., 2017). This suggests either that the slower growing *C. pneumoniae* may be more sensitive to disruptions in iron acquisition or that the faster growing *C. trachomatis* may be more effective at acquiring iron from its host cell and thus better able to resist iron chelation by DFO.

Understanding the molecular basis of *Chlamydia* persistence in response to iron deprivation is important because *Chlamydia* is likely exposed to this persistence trigger during infection. In *C. pneumoniae*, microarray and RT-qPCR analysis demonstrated that *omcB*, a gene associated with outer membrane crosslinking in the EB, and *hctB*, a gene involved in nucleoid condensation during secondary differentiation of RB to EB, were downregulated during iron deprivation (Maurer et al., 2007; Timms et al., 2009). Conversely, a significant increase of transcript level was observed for the stress-response genes *htrA* (21-fold) and *ahpC* (8.5-fold), encoding a periplasmic serine protease and thioredoxin peroxidase, respectively (Timms et al., 2009). In the microarray study by Mäurer and colleagues, the expression of the gene *euo* was unaffected (Maurer et al., 2007). Upregulation of *euo* is well-characterized during IFN-γ-induced persistence, but the apparent lack of change during iron limitation may be due to differences in the normalization method used to analyze the data from this study (i.e., using “housekeeping” genes such as 16*S* rRNA vs genomic DNA levels). In DFO-treated *C. trachomatis* serovar E, transcript data normalized to genomic DNA reported no significant changes in the expression of *euo*, *ompA*, or *omcB* (Dill et al., 2009). However, as noted below, the DFO model of iron-limitation may not reliably limit iron pools to *Chlamydia*, thus these early studies should be interpreted with this caveat.

Unlike persistence mediated by Trp limitation, which can be reliably induced with IFN-γ or a tRNA synthetase inhibitor (Beatty et al., 1994; Hatch and Ouellette, 2020), iron deprivation is dependent on the type of chelator used in the model. DFO is relatively membrane impermeable, and its chelating activity is more efficient in the extracellular environment. Therefore, extensive treatment is necessary to produce sufficient iron starvation for an obligate intracellular pathogen like *Chlamydia* (Pokorzynski et al., 2017). Among chelators which are more membrane permeable, 2,2-bipyridyl (Bpdl) is the most effective at depriving *Chlamydia* of iron to create an iron-deprivation persistent phenotype (Thompson and Carabeo, 2011). In addition to the normalization method used to analyze the data, using different chelators is another source of variability between studies. Results contradicting previous transcriptomic studies were obtained when Bpdl was used instead of DFO. Normalized to genomic DNA, data showed that Bpdl treatment modulates the transcript levels of persistence markers in a similar way to IFN-γ with a high level of *euo* transcripts and a down-regulation of *omcB* expression in a 48 h time course infection (Thompson and Carabeo, 2011). Additionally, the expression of two putative iron-responsive transcripts, *ahpC* and *devB*, is induced by Bpdl treatment and not by DFO (normalization to genomic DNA), despite 30 h of treatment (Dill et al., 2009), which validates the use of Bpdl as a more effective iron chelator for studying effects of iron limitation on *Chlamydia*.

More recently, global transcriptomic studies of iron-deprived *Chlamydia* were conducted with the RNA-sequencing technique. Brinkworth and colleagues demonstrated that, during iron deprivation with Bpdl, 7% of the *C. trachomatis* genome was differentially expressed at the early stage of the development cycle against 8% at the middle stage (Brinkworth et al., 2018). Their data indicated that transcripts for the synthesis of macromolecular precursors (deoxynucleotides, amino acids, charged tRNAs, and acetyl coenzyme A) were upregulated, while transcripts for ABC transporters and translation (genes involved in ribosome assembly, initiation, and termination factors) were downregulated (Brinkworth et al., 2018). The authors hypothesized that this transcriptional response would allow *Chlamydia* to prioritize survival over replication. Recently, a similar approach was used to study the transcriptional profile of the *Chlamydia*-related species *Waddlia chondrophila* exposed or not to iron deprivation. First, *W. chondrophila* also exhibits an aberrant morphology and growth arrest when starved for iron, like *Chlamydia* species. Additionally, the results indicate a transcription profile with a significantly reduced transcript level of genes related to energy production, carbohydrate and amino acid metabolism, and cell wall/envelope biogenesis compared to actively replicating bacteria in the untreated condition (Ardissone et al., 2020). However, three putative TA systems were among the most up-regulated genes upon iron deprivation, suggesting that their activation might be involved in growth arrest in adverse conditions, highlighting a major difference with *Chlamydia* that does not encode TA systems in its genome (Ardissone et al., 2020).

In contrast to IFN-γ-mediated persistence where there is no regulon induced, recent work from the Carabeo laboratory has characterized the function of an iron-responsive transcriptional repressor, YtgR, in the chlamydial response to iron limitation. The *ytg* operon encodes gene products predicted to be necessary for the import of iron into *Chlamydia*, and YtgR is a putative negative regulator of expression in the presence of iron. Preliminary *in vitro* studies of YtgR conducted by the Tan laboratory demonstrated that it binds to an operator region upstream of the *ytg* operon promoter and represses its activity (Akers et al., 2011). Thompson et al. subsequently demonstrated by western blot that the functional YtgR repressor corresponds to a fragment of about 28 kDa produced by the cleavage of the full-length YtgCR, both with proteins samples collected from *C. trachomatis* infected cells or with an overexpression strain of YtgCR in *E. coli*. This 28 kDa C-terminal domain of YtgCR successfully represses the transcription of the *lacZ* reporter gene when driven by the *ytg* promoter, validating its activity as a transcriptional repressor (Thompson et al., 2012).

Recent studies in *C. trachomatis* on the regulation of the *trpBA* operon, coding for the two subunits of the Trp synthase, suggest that a link may exist between iron and Trp limitation (**Figure 3**). Besides binding the *ytg* promoter region, the YtgR repressor, in the presence of iron, can bind to an intergenic region upstream of a *trpBA* alternative promoter and downstream of the regulator *trpR*. This prevents *trpBA* expression by promoting termination of transcripts from the main promoter upstream of *trpR* (**Figure 3**). However, during iron deprivation, YtgR is not able to bind to the alternative promoter, and the Trp synthase can be efficiently produced (Pokorzynski et al., 2019). Moreover, the Trp-codon content of YtgR seems also to play an important role in that regulation. As discussed above, YtgR is processed from its precursor YtgCR protein that possesses a rare triple Trp motif (WWW). Consequently, Trp starvation will negatively impact the translation of the YtgR regulator by promoting the Rho-independent transcription termination of the *ytg* operon with ribosomes stalling at the WWW motif. This alleviates the repression by YtgR as it is no longer translated and further allows *trpBA* expression (see **Figure 3**) (Pokorzynski et al., 2020).

Understanding transcriptional changes that occur during chlamydial persistence is part of the puzzle towards understanding mechanisms involved in the entry or response to conditions that induce chlamydial persistence. Proteomic studies are also very useful tools to investigate how *Chlamydia* responds to iron deprivation and enters persistence. Early proteomic analysis by 2D-PAGE with pulse labeling with [^35^S]methionine in *C. pneumoniae* during iron deprivation-(DFO)-mediated persistence showed increased levels of the OmpA protein whereas OmcB expression was unchanged (Mukhopadhyay et al., 2006). In *C. trachomatis*, between 19 and 25 proteins were induced in response to DFO-mediated iron limitation, however it remains unclear whether most of these proteins participate directly in iron acquisition or homeostasis (Raulston, 1997; Dill et al., 2009). Interestingly, the 60-kDa heat shock protein, Hsp60, was demonstrated to be iron-regulated whereas Hsp60 expression is very stable during IFN-γ-mediated persistence, suggesting that different triggers can use different pathways to induce persistence (Beatty et al., 1994; LaRue et al., 2007). Future proteomic studies using Bpdl as the iron chelating agent could be useful to delineate changes associated with iron limitation and its related persistent phenotype given the known caveats of DFO-mediated iron chelation.

## 5 Persistent infections and *Coxiella burnetii*

### 5.1 Clinical relevance

*Coxiella* infections in humans, referred to as Q fever, occur by the inhalation of *C. burnetii* aerosols generated from infected animals and contaminated environments (Woldehiwet, 2004; Abeykoon et al., 2021). Q fever is a highly transmissible zoonotic disease, and, the more humans are in contact with domestic ruminants/livestock, the more at risk they are of *C. burnetii* infection (Georgiev et al., 2013). Birthing fluids of infected animals are also contaminated by *C. burnetii*, and this pathogen constitutes a real burden in agriculture by causing increased abortion rates and loss of milk production in infected animals (van Asseldonk et al., 2013; Canevari et al., 2018).

A “chronic febrile illness dating back to a proved attack of Q fever” was first used in 1949 to describe a persistent infection in patients (Eldin et al., 2017). As discussed elsewhere and briefly reprised here, Q fever is characterized by two forms. The first one is the acute form with the flu-like symptoms of high fever, severe headache, fatigue, and chills. This first form of the disease is effectively treated by doxycycline even though doxycycline resistance has been observed in a few instances. Sulfamethoxazole-trimethoprim can be used too, and no resistance has been reported to date (Eldin et al., 2017). The second form of the disease is the chronic Q fever, usually treated by the combination of doxycycline and fluoroquinolone (Eldin et al., 2017). Chronic Q fever is a persistent focalized infection, or an infection that is localized to a specific organ site or tissue, that occurs in 1% to 5% of patients, and the most frequent form is endocarditis. The worldwide role of *C. burnetii* as a cause of endocarditis has been recognized in most countries performing systematic serology to identify *C. burnetii* antigens. Like chlamydial infections, 60% of primary *C. burnetii* infections are asymptomatic (Eldin et al., 2017). Similarly, IFN-γ may be a clinically relevant trigger for *C. burnetii* persistence because IFN-γ is detected in the blood of patients suffering from chronic Q fever (Schoffelen et al., 2014).

### 5.2 Biology

*C. burnetii* are small coccobacilli of 0.2 to 0.4 μm wide and 0.4 to 1 μm long. The reservoir is infected livestock animals such as cattle, sheep, and goats, which are infected by a tick vector most frequently belonging to the genera *Ixodes*, *Rhipicephalus*, *Amblyomma*, and *Dermacentor* (Kazar, 2005; Eldin et al., 2017). In mammals, *C. burnetii* replicates inside macrophages within a highly oxidative and acidic vacuole, abbreviated CCV, which has all the characteristics of a terminal phagolysosome: an acidic pH, acid hydrolysates, and cationic peptides (Moffatt et al., 2015).

Analogous to *Chlamydia*, *C. burnetii* has a biphasic developmental cycle including two developmental stages: the large-cell variant (LCV) and the small-cell variant (SCV) (Eldin et al., 2017). The LCV is the replicative form that divides inside the CCV, exhibits dispersed chromatin, and an envelope similar to typical Gram-negative bacteria like *E. coli*. Conversely, the SCV is nonreplicating, metabolically dormant, and has a condensed chromatin. Moreover, a very thick envelope and an unusual internal membrane system, including a high number of peptidoglycan crosslinks, allow the SCV to remain stable in the environment and resist numerous stresses. This enhances resistance to agriculturally related eradication measures (Eldin et al., 2017). *In vivo*, the developmental cycle starts with the entry of a *C. burnetii* SCV into the host cell by endocytosis or phagocytosis. Interestingly, and contrary to chlamydial RBs, the LCV can also be endocytosed to initiate a new infection in cell culture models (Coleman et al., 2004). The CCV is then trafficked along the endolysosomal pathway and acquires the characteristics of a phagolysosome during maturation of the vacuole (Howe et al., 2010). During this maturation, the CCV undergoes vacuole acidification, and lysosome-associated membrane proteins (LAMPs) are recruited to the CCV membrane (Howe et al., 2010). Using a Dot/Icm type 4B secretion system, *C. burnetii* translocates effector proteins into the host cell (Newton et al., 2013). These effector proteins are involved in modification of the CCV to enable *Coxiella* replication and vesicular trafficking to the CCV to acquire nutrients (Beare et al., 2011; Carey et al., 2011; Larson et al., 2016). SCVs are converted into LCVs under the effect of the acidic pH of the phagolysosome, which activates *C. burnetii* metabolism and differentiation (Hackstadt and Williams, 1981). Following differentiation, LCVs replicate exponentially before entering stationary phase. *C. burnetii* growth leads to expansion of the CCV *via* a pathogen-derived process that recruits cellular vesicles to fuse with the vacuole membrane (Howe et al., 2003). Finally, SCVs appear in significant numbers during the stationary phase *via* condensation of the LCVs and are released after cell lysis (Coleman et al., 2004).

### 5.3 Persistence

As with model organisms, *C. burnetii* persistence is characterized by an inhibition of *C. burnetii* replication and growth arrest, which is reversed by the removal of the persistence inducer. Whether the organisms remain infectious during persistence-inducing conditions is not clear. Further, there is relatively little known about the molecular mechanisms of *C. burnetii* persistence. Diverse stimuli can induce *C. burnetii* persistence such as treatment with IFN-γ or TNF-α cytokines, the synthetic nitric oxide donor 2,2′-(hydroxynitrosohydrazino)bis-ethanamine (DETA/NONOate) (Howe et al., 2002), and iron deprivation (Sanchez and Omsland, 2020). In murine L-929 cells infected by *C. burnetii*, IFN-γ and TNF-α treatment induces increased expression of the nitric oxide synthase iNOS, suggesting a contribution of this enzyme to the *Coxiella* persistence state (Howe et al., 2002). Further, these data indicate that oxidative stress more generally could be a trigger of *Coxiella* persistence. Microscopic analysis of infected cells revealed that nitric oxide (either cytokine induced, or donor derived) blocks the maturation of the large CCV. Indeed, exposure of infected cells to nitric oxide resulted in the formation of multiple small, acidic CCVs usually containing only one *C. burnetii* bacterium. Furthermore, addition of the iNOS inhibitor S-methylisothiourea (SMT) to the culture medium of cytokine-treated murine cells allows the fusion of the small CCVs to form a large vacuole harboring multiple replicating *C. burnetii* bacteria. Of note, human cells do not produce high levels of iNOS in response to IFN-γ (Zhang et al., 1996), suggesting that effects related to IFN-γ and/or TNF-α are more likely related to other factors like IDO activity or autophagy.

In many bacteria, the protein Fur (ferric uptake regulator) is the transcriptional regulator of genes coding for iron acquisition proteins (Xiong et al., 2000; Fillat, 2014; Seo et al., 2014). Indeed, during iron deprivation, the expression of the Fur regulon allows bacteria to increase iron uptake. Previous work tried to identify a Fur regulon in *C. burnetii* because a *fur* homologue is present in its genome (**Table 1**). The *C. burnetii fur* gene functionally complements an *E. coli fur* deletion strain. In the same study, a putative ferrous iron uptake transporter, FeoAB, was identified as a member of the Fur regulon in *C. burnetii* (Briggs et al., 2008). This transporter functions in ferrous iron transport (Fe^2+^) in *Legionella pneumophila*, a close phylogenetic relative of *C. burnetii* (Robey and Cianciotto, 2002). The presence of this putative transporter in *C. burnetii* genomes suggests that Fe^2+^ is the natural source of iron used by *C. burnetii*. In a recent report, Sanchez & Omsland studied the effect of iron deprivation on *C. burnetii* in a host cell-free culture model. The development of citrate cysteine ACCM-1 and ACCM-2 axenic media allowing *C. burnetii* growth outside of a host cell has facilitated this type of study in *C. burnetii* (Sandoz et al., 2014). In ACCM-2 medium supplemented with a range of iron sulfate FeSO_4_ (from 1 to 250 μM), results indicate that *C. burnetii* tolerates molecular iron over a broad concentration range and undergoes loss of viability upon iron deprivation (Sanchez and Omsland, 2020). Moreover, chelation of host iron pools by different concentrations of Bpdl treatment for 3 days inhibited *C. burnetii* replication during infection of Vero cells. Other experiments are necessary to determine if iron-replete medium rescues *Coxiella* replication in the host cell, which would be consistent with persistence (Sanchez and Omsland, 2020).

Transcriptional control of gene expression, generally, is regulated by sigma factors that confer promoter specific initiation of transcription by RNA polymerase. Some sigma factors are expressed to respond to specific growth conditions encountered by bacteria. For example, in many bacteria, RpoS regulates gene expression during the stationary phase of growth and other stress conditions including nutrient starvation (Gottesman, 2019). Seshadri & Samuel (2001) demonstrated that the expression of *C. burnetii rpoS* successfully activated the expression of *lacZ*, as measured by a 5-fold increase in β-galactosidase activity, under carbon starvation in an *E. coli* strain deleted for its *rpoS* gene. Then, using protein samples collected from *C. burnetii* infected cells, they demonstrated by western blot that RpoS was highly expressed in the LCV form, but not in the SCV form (Seshadri and Samuel, 2001). This was unexpected because LCVs are present during the exponential phase of growth, when nutrients are abundant and allow rapid multiplication of *C. burnetii*, whereas SCVs appear in the stationary phase of growth when less nutrient availability is more likely to induce RpoS expression. More recently, Dresler and colleagues aimed to quantify by mass spectrometry proteome changes in the *C. burnetii* Nine Mile phase I and II isolates (NMI and NMII, respectively) grown in different axenic media corresponding to different growth conditions and in mouse fibroblasts. Unlike the Seshadri & Samuel study, conducted before the development of axenic media, they found that the level of the transcriptional regulatory proteins RpoS and SpoT and translational regulatory proteins as CsrA2, UspA1, and UspA2 was increased for the NMI isolate in ACCM-D, a defined medium mimicking the stationary phase of growth. Moreover, in the NMI isolate, transcriptional regulators of the exponential phase of growth, Fis and RpoD, were downregulated (Dresler et al., 2019). Interestingly, RpoS seems to be a major regulator involved in the morphological differentiation between the LCV and the SCV forms of *C. burnetii*. Using the ACCM-D medium, RNA sequencing was performed in wild-type and *rpoS* null mutant (Δ*rpoS*) *C. burnetii* NMII strain LCVs and SCVs to identify the *rpoS* regulon during nutrient starvation. In this study, the *rpoS* regulon as well as the RpoS binding site on the promoter sequences were revealed (Moormeier et al., 2019). At the transcriptional level, 25 genes were significantly dysregulated in the Δ*rpoS* SCVs. Liquid chromatography with tandem mass spectrometry on whole cell bacterial lysates of SCVs from wild-type and Δ*rpoS* strains were performed to study proteomic changes. They observed a correlation with the RNA sequencing results and concluded that the alternative sigma factor RpoS positively regulated the expression of many genes involved in SCV development including genes involved in oxidative stress response, arginine transport, peptidoglycan remodeling, and synthesis of the SCV-specific protein ScvA. On the contrary, RpoS downregulated the expression of Dot/Icm T4BSS genes (Moormeier et al., 2019).

How *C. burnetii* responds to environmental stresses or enters persistence is not completely understood. Given that *C. burnetii* possesses functional homologs of the genes *relA* and *spoT* responsible for ppGpp production, a feature unique to this obligate intracellular pathogen, it could rely on functional stringent response to cope with nutrient starvation. As mentioned above, *C. burnetii* is auxotrophic for several amino acids, including Trp, thus amino acid starvation (and uncharged tRNAs binding in the A site of the ribosome) is likely to trigger the stringent response in this organism. In *L. pneumophila*, the stringent response and RpoS control the developmental cycle and especially the differentiation into the transmissive form (Dalebroux et al., 2009). Under nutrient deprivation, the ppGpp synthetase RelA produces ppGpp which then induces RpoS synthesis. Finally, RpoS coordinates *L. pneumophila* differentiation (Dalebroux et al., 2010). The *C. burnetii* LCV-to-SCV differentiation could be similar to that of *L. pneumophila*, but the specific involvement of the stringent response in persister formation has not been evaluated.

## 6 Persistence and the Rickettsiales order

The Rickettsiales order comprises two families of very diverse Gram-negative and obligate intracellular bacteria transmitted to human and animals by ticks or mites. The *Anaplasmataceae* family includes pathogenic species for humans, *Anaplasma phagocytophilum* and *Ehrlichia chaffeensis*. The *Rickettsiaceae* family includes a group of typhus-causing pathogens comprising *Rickettsia* spp. and *Orientia tsutsugamushi*. Bacteria in the genus *Rickettsia* are classified in several groups: the spotted fever group (*Rickettsia rickettsia*, *Rickettsia conorii*, *Rickettsia africae* and *Rickettsia parkeri*), the typhus group (*Rickettsia prowazekii* and *Rickettsia typhi*), the transitional group (*Rickettsia felis* and *Rickettsia akari*) and the ancestral group, which are not associated with causing disease in humans or animal reservoirs (*Rickettsia canadensis* and *Rickettsia bellii*) (Salje, 2021). Rickettsial pathogens are inoculated into the blood or skin of humans through the saliva or feces of feeding arthropods and are disseminated through the body *via* the blood and/or lymphatic system. The tropism to mammalian cells varies between species, and includes neutrophils (*A. phagocytophilum*), monocytes/macrophages (*E. chaffeensis*, *O. tsutsugamushi*), endothelial cells (*Rickettsia* spp., *O. tsutsugamushi*), and dendritic cells (*O. tsutsugamushi*) (Salje, 2021).

### 6.1 Clinical relevance

Pathogens from the *Anaplasmataceae* family can cause widespread infections in human and animals. Human pathogens include *A. phagocytophilum* and *E. chaffeensis*, responsible for the human granulocytotropic anaplasmosis (HGA) and the human monocytotropic ehrlichiosis (HME) diseases, respectively. The clinical manifestations of HGA include mild febrile illness, severe fever, chill, headache, and myalgia; and they are often accompanied by thrombocytopenia, leukopenia, and elevated serum levels of hepatic enzymes (Rar et al., 2021). Rare cases also reported meningitis and encephalitis. The severity of infections seems to vary depending on the geographical area with more severe infections in the USA than in European countries (Rar et al., 2021). Symptoms of HME are not specific and range from influenza-like symptoms to life-threatening disease (Torres-Escobar et al., 2021). Chronic infections were observed for *A. phagocytophilum* in infected animals like horses, sheep, dogs, or lambs. Positive diagnosis of chronic infection involves detection of *A. phagocytophilum* DNA in blood samples by PCR over long periods of time (Franzen et al., 2009; Thomas et al., 2012; Hovius et al., 2018). Concerning *E. chaffeensis*, chronic infections have been reported in the white-tailed deer (reservoir), humans, and dogs (incidental hosts) (Breitschwerdt et al., 2014; Nair et al., 2014). In white-tailed deer and dogs, *E. chaffeensis* persistence was assessed by detection of bacterial DNA in blood samples, by PCR, up to 41-63 days post infection (Nair et al., 2014). In contrast, long-term chronic infections in humans have not been reported (Bakken and Dumler, 2015).

The symptoms of rickettsioses usually begin 5-14 days post inoculation *via* tick bite, exposure to feces of infected lice and fleas (*Rickettsia* spp.), or the bite of chiggers and mites (*O. tsutsugamushi*) (Osterloh, 2020). The symptoms of human rickettsial diseases are non-specific and present with headache, fever, myalgia, and sometimes a rash or an eschar in scrub typhus (*O. tsutsugamushi*) and some spotted fever diseases. In the United States, Rocky Mountain spotted fever caused by *R. rickettsia* infection is the most severe and frequently reported rickettsial disease. Rickettsial species are not susceptible to many classes of antibiotics. Tetracyclines, macrolides, chloramphenicol, or rifamycins are the preferred drug classes to treat rickettsial infections. Disease severity is variable and can be life-threatening due to misdiagnosis or treatment with an inappropriate drug (Osterloh, 2020). Despite the effectiveness of early antibiotic treatment, the fatality rates for rickettsial pathogens range from 1% to 10% (Ellison et al., 2009). Persistent infections after antibiotic therapy have been observed for infections by *R. rickettsia* (Parker et al., 1954; Hove and Walker, 1995), *R. prowazekii* (Bechah et al., 2010), and assumed for *R. typhi* (detection 3 to 4 months after infection in the brain of infected mice in a model of *R. typhi* persistent infection) (Osterloh et al., 2016).

Despite its high incidence and severity, *O. tsutsugamushi* is less studied than other rickettsial species such as *R. prowazekii*, *R. conorii*, and *R. rickettsia*. *O. tsutsugamushi* is the causative agent of the severe human scrub typhus disease, which affects about 1 million people per year in Asia, Oceania, and Northern Australia (Atwal et al., 2017; Salje, 2021). Under-recognition and under-reporting are a major public health issue in scrub typhus due to difficulties with correct diagnosis and the lack of knowledge amongst clinicians (Mika-Gospodorz et al., 2020). *O. tsutsugamushi* is also able to persist in cell culture models of infection (Kim et al., 1999) or in mouse models of infection (Shirai et al., 1979; Kee et al., 1994; Chung et al., 2012). Cases of persistent *O. tsutsugamushi* infections associated with early and late relapses have been reported (Chung et al., 2012).

### 6.2 Biology

*A. phagocytophilum* and *E. chaffeensis* reside within a host-derived vacuole and possess a biphasic developmental cycle that alternates between a replicative intracellular form called the reticulate cell and an infectious extracellular form called the dense-core cell. Vacuoles containing multiple reticulate cells are called morulae because of the resemblance to mulberries (Salje, 2021). The differentiation step is well-regulated, and the two-component system transcriptional regulator CtrA may be involved in differentiation regulation in *E. chaffeensis*. This two-component regulator is conserved among the Rickettsiales (Salje, 2021). After the first differentiation, the reticulate cells divide by binary fission for ~48 h. At that time, the vacuole contains several hundred bacteria that undergo secondary differentiation to form the mature dense-core cells. In the case of *A. phagocytophilum*, release of infectious organisms occurs through exocytosis of membrane-bound bacterial vacuoles or by cell lysis around 72 h post-infection (Scherler et al., 2018).

*Rickettsia* spp. replicate in endothelial cells of blood vessels and major organs or macrophages in the case of *R. conorii*, *R. parkeri*, and *R. akari* (Salje, 2021). After entry into the host cell, rickettsial phospholipase degrades the phagosomal membrane, allowing bacteria to replicate freely in the host cell cytoplasm (Renesto et al., 2003; Rahman et al., 2013). *Rickettsia* spp. from the spotted fever group move directly into adjacent cells by a mechanism dependent on actin polymerization and with the Sca4 protein (Heinzen, 2003; Haglund et al., 2010; Reed et al., 2014; Lamason et al., 2016). *Rickettsia* spp. from the typhus group replicate throughout the cytoplasm of the host cell (100 bacteria per cell or more) until cell lysis occurs to release the intracellular bacteria (Silverman et al., 1980; Salje, 2021). Actin-based motility has not been demonstrated for *R. prowazekii*, and *R. typhi* produces small and rare actin tails conferring limited motility (Teysseire et al., 1992). The non-motility of *R. prowazekii* and the poor motility of *R. typhi* may be explained by the absence of the *rickA* gene in their genome (McLeod et al., 2004). The product of the *rickA* gene encodes the bacterial surface protein RickA known to interact with the host Arp2/3 complex to induce actin polymerization into short and curved tails (Gouin et al., 2004). In a similar way to the other *Rickettsiaceae*, *O. tsutsugamushi* replicates directly in the cytoplasm of the host cell using a specific microtubule-driven mode of motility (Kim et al., 2001; Wongsantichon et al., 2020). It was previously observed that *O. tsutsugamushi* is able to bud off the host cell, and Atwal et al. recently demonstrated that the budded *O. tsutsugamushi* (in a host cell membrane envelope) is in a distinct developmental stage compared to the intracellular form (Rikihisa and Ito, 1980; Atwal et al., 2022). Indeed, intracellular and extracellular *O. tsutsugamushi* are molecularly different, and the extracellular *O. tsutsugamushi* expresses higher levels of the stress proteins SpoT and RpoH (heat shock sigma factor) and of the surface protein ScaC. The two forms differ also in their peptidoglycan content as the extracellular *O. tsutsugamushi* exhibits a lower level of peptidoglycan, suggesting a higher sensitivity of this form to physical and osmotic stressors.

### 6.3 Persistence

It remains unclear whether a true persister state is established in *Rickettsiaceae* or whether a low-level chronic infection is maintained. For instance, no persister phenotype has been described among the *Rickettsiaceae*. However, rickettsial antigens including bacterial DNA can be detected in hosts over several months, suggesting chronic infection. Chronic *Anaplasma* spp. infections of animals may be linked to antigenic variation of the major surface proteins (MSPs) to produce new antigenic variants (Brayton et al., 2001; Brayton et al., 2002; Barbet et al., 2003). Random mutagenesis using transposon libraries identified bacterial genes involved in pathogenesis of *Ehrlichia*. For example, the gene ECH_0660 was required for *Ehrlichia* growth in deer (Cheng et al., 2013). Additional studies revealed ECH_0660-dependent upregulation of a cluster of seven genes during iron or zinc deprivation (Torres-Escobar et al., 2021). A transposon mutagenesis library of *E. chaffeensis* by Wang and colleagues identified mutants for the genes ECH_0837 (coding for the metal ion binding protein MiaB) and ECH_1144 (coding for an outer membrane protein) with a significantly slower growth than wild-type *E. chaffeensis* in the macrophage cell line DH82 (Wang et al., 2020). Despite these reports, it is unclear if iron or zinc deprivation impacts *E. chaffeensis* persistence in the host. A broader study using *in vivo* canine infection models to identify *Ehrlichia* determinants of long-term bacterial maintenance yielded 13 independent *E. chaffeensis* mutants. These mutants remained detectable for 8 weeks. Interestingly, ECH_0837 and ECH_1144 were not identified in this study, indicating fundamental differences in chronic or persistent infections of cell lines compared to *in vivo* models (Wang et al., 2020). Further studies will reveal the response mechanisms of *Anaplasma* and *Ehrlichia* to nutrient limitations in the absence of a stringent response and other factors (**Table 1**).

Phylogenetic analyses identified type II TA systems of the VapBC family in the genomes from the *Rickettsia* genus, as well as other putative TA genes (Gillespie et al., 2008; Audoly et al., 2011), but their function as TA systems has been largely unexplored. *R. conorii* expresses a VapBC TA system, and both genes were upregulated during *in vitro* exposure to 50 mg/L of the fluoroquinolone ciprofloxacin (Botelho-Nevers et al., 2012). Furthermore, *vapB* and *vapC* genes are among the most highly expressed genes during *R. conorii* infection of ticks and host cells (Narra et al., 2020). Even less is known about *O. tsutsugamushi* mechanisms of host cell invasion, pathogenesis, or persistence. *O. tsutsugamushi* is auxotrophic for the aromatic amino acids and histidine, suggesting nutrient starvation could be a potential trigger for persistence (Rodino et al., 2018). Interestingly, a higher level of L-kynurenine was detected in the serum of patients with scrub typhus (Prachason et al., 2012). L-kynurenine is the Trp degradation product of the IDO enzyme that is produced in infected human macrophages and epithelial cells stimulated with IFN-γ. In a cell culture model of *O. tsutsugamushi* THP-1 infected cells, Prachason et al. showed that treating the infected cells with IFN-γ resulted in growth inhibition. This was reversed by the addition of high doses of Trp in the cell culture medium, suggesting that Trp depletion is the principal anti-*Orientia* effector of IFN-γ, with a low-Trp environment triggering a slow-growth phenotype in *O. tsutsugamushi* (Prachason et al., 2012). Currently, no genetic tools are available to study *O. tsutsugamushi*. Experimentally validated fluorescent probes to label *O. tsutsugamushi* were recently developed, allowing live cell imaging experiments to study its host cell infection cycle in detail (Atwal et al., 2016). Persistence studies at the molecular level among species of the *Rickettsiaceae* family are progressing slowly due to the limited genetic tools available. Detailed understanding of the pathogenic mechanisms, including establishment of persistence will depend on a robust genetic toolkit.

## 7 Concluding remarks

Persistence is a common phenomenon to bacteria in which it has been studied, and this is likely to include the obligate intracellular bacteria. A key parameter of all persisters is their reduced or stalled growth rate. Whereas in “model” organisms a critical function for TA systems has been suggested, most obligate intracellular bacteria lack these systems. Nonetheless, their absolute dependence on the host cell renders them highly susceptible to disruptions in nutrient supplies often elicited by immune responses against these pathogens. The phenotypic consequence of this, as exemplified by *Chlamydia*, is stalled growth that effectively mimics a persister phenotype. This is particularly critical for obligate intracellular bacteria. Persistence allows these bacteria to maintain a long-term interaction with their mammalian host cell under adverse conditions, which is essential to their viability.

Current knowledge about obligate intracellular bacterial persistence is variable depending on the availability of genetic tools in a particular organism to study persistence mechanisms at the molecular level. However, the stringent response and TA systems are not major factors in establishing persistence of obligate intracellular bacteria, demonstrated by the fact that most species lack TA genes or *relA/spoT* genes involved in ppGpp synthesis or both. Considering their unique growth adaptations and tissue tropism in the host, persistence triggers vary and may induce different stress responses ultimately leading to a persistence phenotype. Therefore, it is likely that new strategies for inducing persistence may be revealed from studying these organisms. Alternatively, the conservation of the *obgE* gene in obligate intracellular bacteria, as well as recent studies highlighting the involvement of that GTPase in *E. coli* persistence, suggests all organisms may share a means by which they become persistent. Clearly, further work in these areas is required to better understand how, why, and under what circumstances obligate intracellular bacteria persist *in vivo* and how their persistence impacts the host at a molecular and physiological level.

## Author contributions

CR wrote the initial draft, ER and SO edited and provided guidance and funding support. All authors contributed to the article and approved the submitted version.
